# Drawing a materials map with an autoencoder for lithium ionic conductors

**DOI:** 10.1038/s41598-023-43921-1

**Published:** 2023-10-05

**Authors:** Yudai Yamaguchi, Taruto Atsumi, Kenta Kanamori, Naoto Tanibata, Hayami Takeda, Masanobu Nakayama, Masayuki Karasuyama, Ichiro Takeuchi

**Affiliations:** 1 Department of Advanced Ceramics, Nagoya Institute of Technology, Gokiso, Showa, Nagoya, Aichi 466-8555 Japan; 2 Department of Computer Science, Nagoya Institute of Technology, Gokiso-cho, Showa-ku, Nagoya, Aichi 466-8555 Japan; 3 RIKEN Center for Advanced Intelligence Project, 1-4-1 Nihonbashi, Chuo-ku, Tokyo, 103-0027 Japan; 4 Faculty of Engineering, Nagoya University, Furo-cho, Chikusa-ku, Nagoya, Aichi 464-8601 Japan

**Keywords:** Theory and computation, Batteries, Cheminformatics

## Abstract

Efforts to optimize known materials and enhance their performance are ongoing, driven by the advancements resulting from the discovery of novel functional materials. Traditionally, the search for and optimization of functional materials has relied on the experience and intuition of specialized researchers. However, materials informatics (MI), which integrates materials data and machine learning, has frequently been used to realize systematic and efficient materials exploration without depending on manual tasks. Nonetheless, the discovery of new materials using MI remains challenging. In this study, we propose a method for the discovery of materials outside the scope of existing databases by combining MI with the experience and intuition of researchers. Specifically, we designed a two-dimensional map that plots known materials data based on their composition and structure, facilitating researchers’ intuitive search for new materials. The materials map was implemented using an autoencoder-based neural network. We focused on the conductivity of 708 lithium oxide materials and considered the correlation with migration energy (ME), an index of lithium-ion conductivity. The distribution of existing data reflected in the materials map can contribute to the development of new lithium-ion conductive materials by enhancing the experience and intuition of material researchers.

## Introduction

Research in the field of material informatics (MI) has become increasingly active with recent developments in ceramic material databases and computational performance (CPUs and GPUs). MI combines informatics with materials exploration to accelerate the search for new functional materials. Examples of inorganic materials databases, such as the Materials Project^1^ and the Inorganic Crystal Structure Database (ICSD)^2^, contain a vast amount of crystal structure data points and material properties estimated using computational simulations. These databases have facilitated many studies in materials science using high-throughput calculations, such as first-principles and force-field calculations^3–5^. By applying MI to these databases or via high-throughput studies, researchers can organize complex relationships between the compositions, structures, and physical properties of materials, facilitating the more efficient search of useful materials. The process typically involves converting material data into descriptors *x*_*i*_, and learning a prediction function that yields the desired physical properties as the objective variable *Y*^6^. Descriptors numerically represent crystal structures and serve as an interface between crystal structure and material property data. Researchers (users) can propose candidate new materials, convert them into descriptors, and predict their physical properties instantly^4,7–9^. By identifying promising materials in advance, experimental costs can be significantly reduced. Furthermore, analyzing the constructed prediction functions using methods such as importance variable analysis may provide chemical semantic interpretations of the descriptors^10,11^. However, existing methods face challenges in identifying unknown materials that are not registered in databases. Inorganic crystalline material descriptors contain information derived from composition, crystal structure and other characteristics. Although it is easy to generate descriptors from conventional crystal structure descriptions, such as the lattice parameters and fractional coordinates of ions, reconstruction of crystal structures from those descriptors remains challenging. Consequently, the MI approach, which uses such descriptors, cannot predict the physical properties of undiscovered compounds—i.e., non-registered compounds—in the material database. Attempts have been made to evaluate phase stability and other physical properties by constructing machine learning models using only compositional information, without using structure-derived descriptors^12–14^. A composition and its descriptors are reversible, and users can easily obtain evaluation results by simply considering their compositions. However, the accuracy of evaluation results is greatly reduced as material properties are highly dependent on both composition and crystal structure information. Nevertheless, there is significant potential in areas outside the scope of existing material databases. According to a prior report^15^, it is estimated that only 16% of inorganic compounds have been discovered in ternary systems and merely 0.6% in quaternary systems. In another paper^16^, it was estimated that, in compositional combinations considering charge neutrality, there were 30 million cases in ternary systems and 30 billion cases in quaternary systems. This indicates that novel functional materials are likely hidden in the vast space of undiscovered materials, necessitating an efficient exploration approach. The conventional MI approach alone is inefficient for determining the compositions and structures of undiscovered materials with desired properties. To support specialized researchers and enhance their intuition, this study introduces the concept of a materials map, which visualizes existing datasets in a two-dimensional space. Even today, numerous groundbreaking material discoveries are believed to heavily rely on the intuition and expertise of exceptional specialists. Therefore, the development of MI-derived tools that can effectively and efficiently support these experts is expected to accelerate the discovery process.

One challenge experienced by researchers when using the MI approach arises from the utilization of high-dimensional descriptors as inputs for the MI scheme. To optimize the intuition and perspective of researchers, this study introduces the concept of a *materials map*, which transforms existing datasets into a two-dimensional space. This map evaluates materials based on two intuitive scales: chemical composition and crystal structure. Consequently, it enables the specification of the search range for known materials while simultaneously identifying unexplored materials. Notably, 2D materials maps using fundamental properties, such as electronegativity and ionic radius, have been proposed in several prior studies^17,18^. However, the experience of researchers invaluable when selecting relevant coordinates since conventional materials maps only consider specific indices. Therefore, in this study, the descriptors were dimensionally reduced using an autoencoder, a type of deep learning model, while retaining maximum information related to composition and structure. Furthermore, by applying a neural network structure associated with the target properties, we developed a materials map tailored to user-specified target properties.

## Dataset

To validate the materials mapping method developed in this study, we present an application example focusing on candidate solid electrolyte materials in all-solid-state batteries, which are attracting attention as a next-generation energy source. One crucial property of solid electrolyte materials is their high Li-ion conductivity. We used a dataset comprising the data of 708 conductivity simulations of Li–O-based inorganic solid materials, which we reported previously^19^. All compounds were selected from the Materials Project database and matched two conditions: (1) inclusion of Li and O, and (2) incorporation of 25 elements (Mg, Ca, Sr, Ba, Sc, Y, La, Ti, Zr, Hf, Nb, Ta, Zn, B, Al, Ga, In, C, Si, Ge, Sn, P, As, Sb, S). For samples meeting these conditions, the migration energy (ME, denoted as E_mig_ [eV]) of lithium-ion diffusion in the solids was calculated using an automated ion conductivity evaluation algorithm based on the Bond Valence Force Field (BVFF)^20^ method, where smaller values of E_mig_ indicate a smaller barrier to Li-ion diffusion and thus better conductivity. The compositional information considered in this study includes the chemical composition ratio and various properties of constituent elements (atomic number; atomic weight; electronegativity; melting point in the metallic state; atomic/ion/covalent bond radii; number of s-, p-, d-, and f-electrons; and Mendeleyev number) expressed in histogram form^21^. Three types of structural information were employed: the Radius Distribution Function (RDF), the Angular Distribution Function (ADF), and the Voronoi diagram. Compositional and structural descriptors were represented by numerical sets of 1782 and 858 dimensions, respectively. All descriptors and E_mig_ values were scaled by the maximum value for each type (compositional descriptors, RDF, ADF, Voronoi diagrams, and E_mig_) and converted to a range of 0 to 1. All data tables are available in the Supporting Information.

## Methods

**Figure 1:**
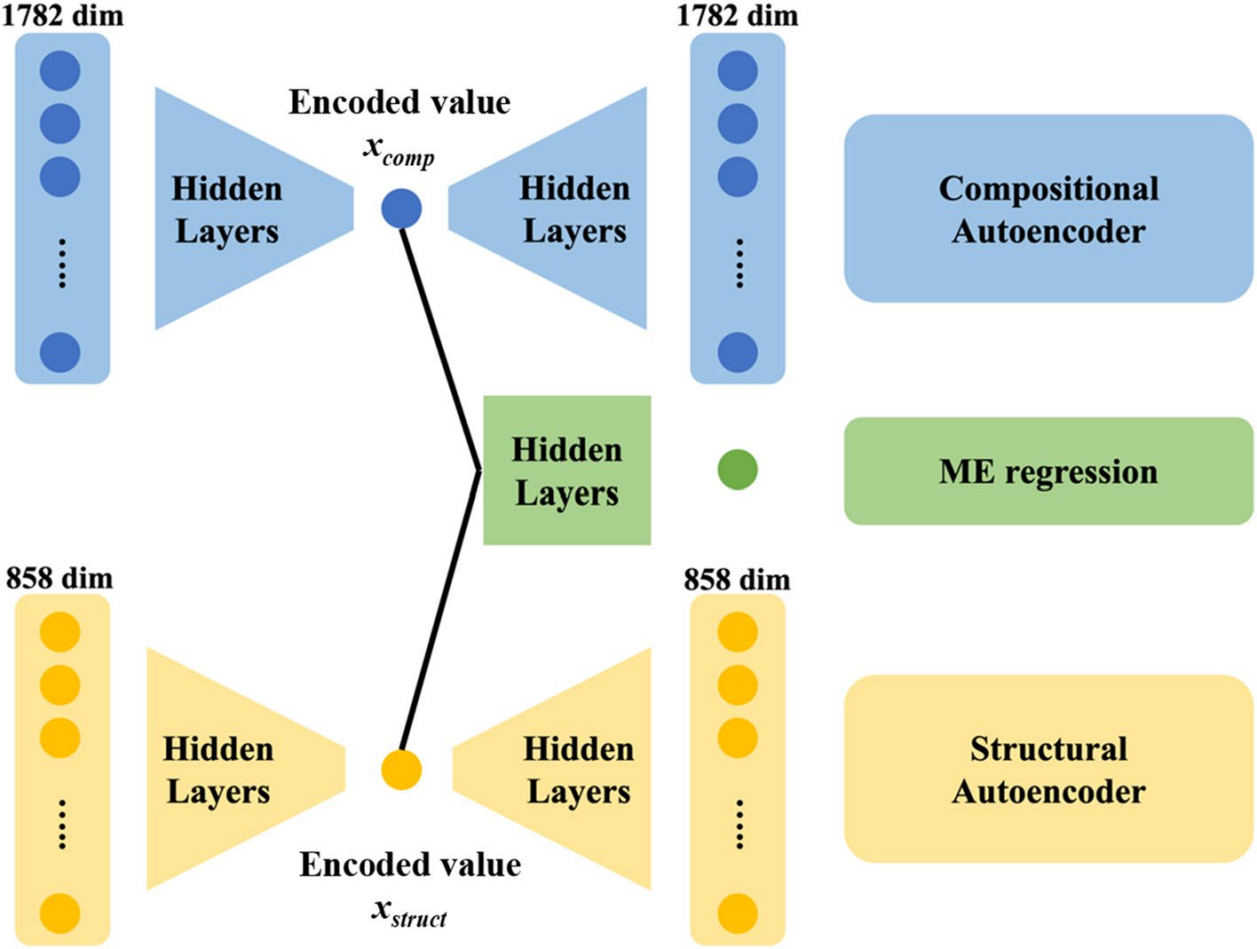
Autoencoder architecture for the materials map. Compositional and structural descriptors are condensed into a one-dimensional format, producing encoded values, denoted as *x*_*comp*_ and *x*_*struct*_, which are linked to the migration energy (ME) as the target objective.

In this study, an autoencoder was used to compress each of the compositional and structural descriptors into one dimension each. The autoencoder^22^ is a neural network model with specific constraints on the number of neurons and layer shapes. It is an unsupervised machine learning technique that can effectively reduce the dimensionality of input information. Upon receiving an *n*-dimensional input, the number of neurons (dimensions) is reduced (encoded) to the user-specified dimensionality through the application of fully connected layers and activation functions (encoded values), and the encoded values are then expanded (decoded) to ensure that the output’s dimensionality matches that of the input (decoded values). When the autoencoder successfully reproduces the input descriptors in its outputs, the encoded values effectively capture all the information from the original descriptors. In this study, the compositional and structural descriptors were compressed to one dimension each and then used to generate a two-dimensional map based on the compositional and structural axes. Furthermore, an intermediate layer was introduced to learn the relationship between ME and the 2D information in the coding layer, allowing for the creation of a materials map associated with ME. A schematic of the neural network model used in this study is presented in Fig. 1. To optimize the model weights, loss functions were defined as Eqs. (1) and (2), corresponding to minimization of the mean squared error (MSE).

1 $$\begin{array}{c}{Loss}_{comp}= \frac{1}{n}\sum \limits_{i=1}^{n}\sum\limits_{j=1}^{d}{\left({Out \,comp}_{i, j}-{In \,comp}_{i, j}\right)}^{2},\end{array}$$

2 $$\begin{array}{c}{Loss}_{str}= \frac{1}{n}\sum \limits_{i=1}^{n}\sum_{j=1}^{d}\limits{\left({Out \,str}_{i, j}-{In \,str}_{i, j}\right)}^{2},\end{array}$$

where *n* and *d* denote the number of samples and descriptors respectively.

In addition, the two encoded variables derived from the composition and structure descriptors are associated with the lithium ions’ ME using a network structure, as illustrated in Fig. 1. The loss function is defined by Eq. (3), which also considers the MSE.

3 $$\begin{array}{c}{Loss}_{ME}= \frac{1}{n}\sum\limits_{i=1}^{n}{\left({Pred}_{i}-{True}_{i}\right)}^{2}.\end{array}$$

The loss function for the entire model is defined as the sum of *Loss*_*comp*_, *Loss*_*str*_, and *Loss*_*ME*_ multiplied by the weight factor *W* (Eq. 4).

4 $$\begin{array}{c}{Loss}_{all}={Loss}_{comp}+{Loss}_{str}+W\times {Loss}_{ME}.\end{array}$$

The weight factor, *W*, controls the priority assigned to ME prediction. A larger *W* prioritizes the accuracy of ME regression, whereas a smaller *W* prioritizes the recovery of compositional and structural descriptors. We examined patterns with *W* = [0, 0.01, 0.1, 1] to balance the accuracy of ME prediction with the reconstruction of input descriptors. The batch size, learning rate, and L2 penalty were set as hyperparameters, and a grid search was conducted to determine the combination that minimized *Loss*_*all*_. The number of epochs was determined when *Loss*_*all*_ did not improve for 80 consecutive optimizations using the Adam optimizer^23^. Hyperparameter tuning resulted in a batch size of 16, learning rate of 0.001, and L2 penalty of 10^−6^. To train the materials map autoencoder, 80% of the data were randomly selected and assigned to the training set, and the remaining 20% were used as test data to verify the generalizability of the model.

## Results and discussion

**Figure 2:**
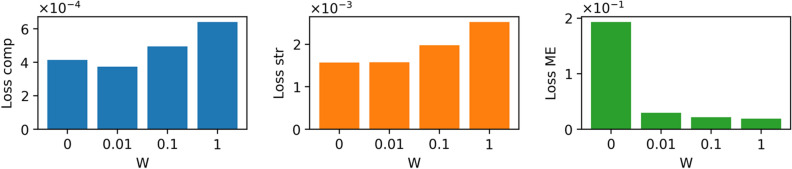
Loss functions of (**a**) compositional descriptors, (**b**) structural descriptors, and (**c**) ME regression, represented as functions of the weight parameter *W*. This parameter governs the trade-off in performance between composition/structure reconstruction and ME prediction.

**Figure 3:**
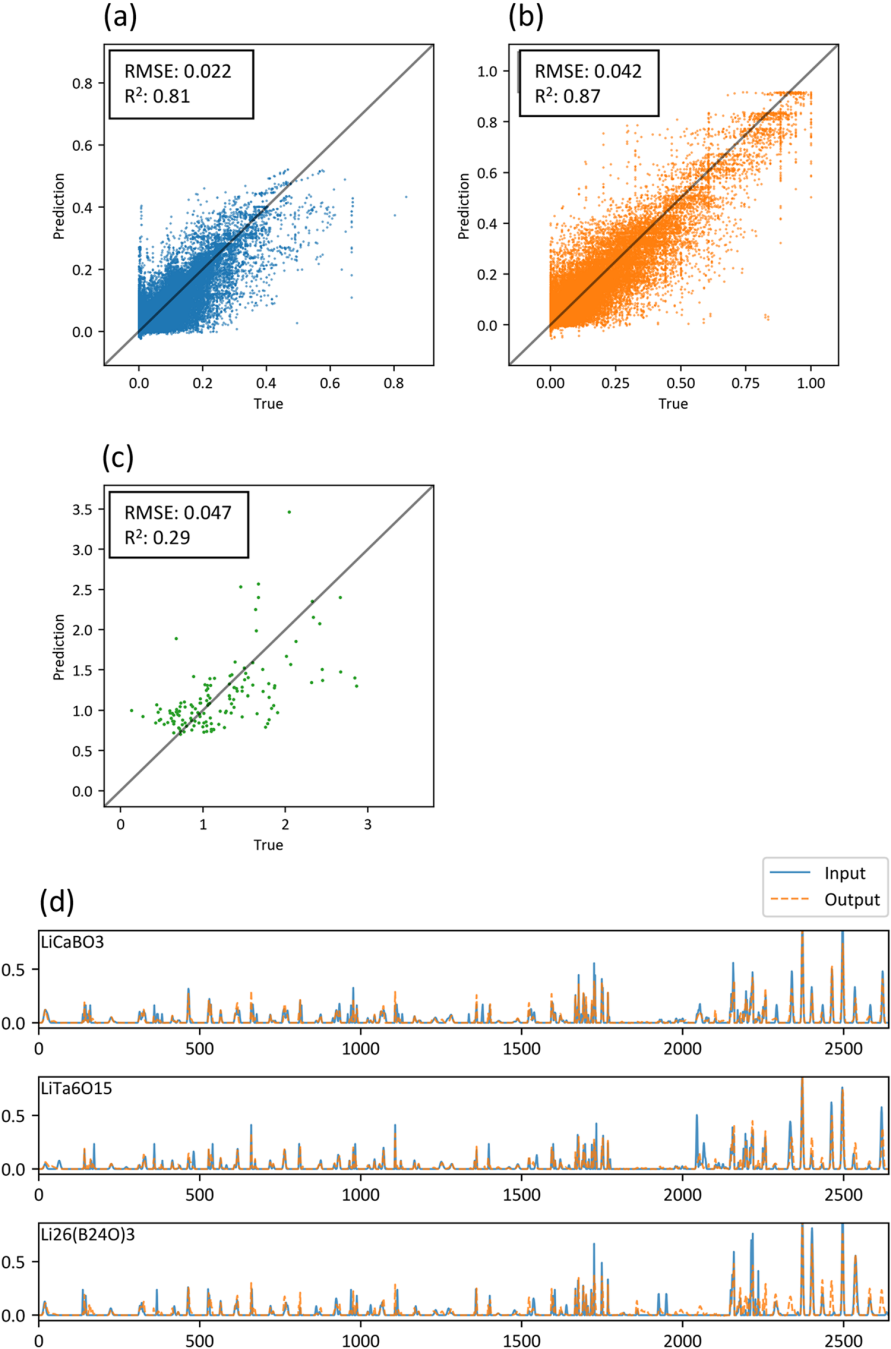
Reconstruction evaluation of compositional/structural descriptors and ME prediction performance of the materials map autoencoder. Diagnosis plots of (**a**) compositional and (**b**) structural descriptors, as well as (**c**) ME predictions, along with (**d–f**) three examples of input and output histogram-descriptors that correspond to the three poorest reconstruction loss functions. Compositional descriptors are represented by numbers 0–1782, while structural descriptors are represented by numbers 1783–2640.

Figure 2 shows values of *Loss*_*comp*_, *Loss*_*str*_, and *Loss*_*ME*_ obtained for different values of *W*, demonstrating a trade-off relationship between the reconstruction of composition and structure descriptors (*Loss*_*comp*_ and *Loss*_*str*_) and the regression of migration energy (*Loss*_*ME*_) as a function of *W*. We adopted a weight factor of *W* = 0.1 to balance the performance of both processes. Figure 3a–c depict diagnostic plots of the test data for composition, structure, and ME regression predictions at W = 0.1. Flattened values for the composition (1782 dimensions) and structural (858 dimensions) descriptors are displayed in Fig. 3a,b. The coefficient of determination, *R*^*2*^ score, exceeded 0.8, indicating the accurate reconstruction of both descriptor types. Figure 3d–f compare the input and output descriptors for the three selected samples that exhibit the largest loss values (*Loss*_*all*_), confirming that the rough shapes of the descriptors were reconstructed. On the other hand, the ME regression (Fig. 3c) exhibits a very low *R*^2^ score of 0.29, which stems from the insufficient prediction power of the original compositional and structural descriptors as reported in our paper^19^.

**Figure 4:**
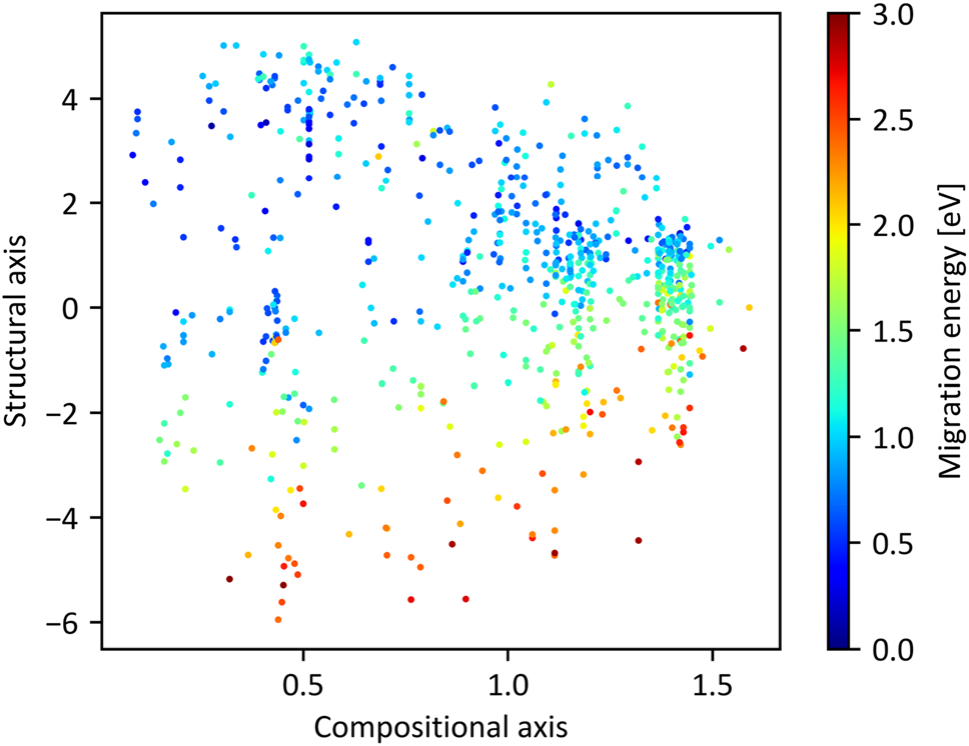
Map for Li-ion conductive materials in the Li–O system. The horizontal and vertical axes correspond to the compressed compositional and structural descriptors, respectively, achieved through the autoencoder approach.

Figure 4 shows a map of the constructed materials, with the horizontal axis obtained by compressing the compositional descriptor (1782 dimensions) to one dimension, and a vertical axis likewise obtained by compressing the structural descriptor (858 dimensions) to one dimension. Because each axis was produced by an autoencoder, no units or clear chemical interpretation are present. The colors of the plots in the figure correspond to the MEs of lithium ions, with larger and smaller values represented in dark red and dark blue, respectively. Note that the research and development of battery materials requires high lithium-ion conductivities, which correspond to materials with a low ME. Encoded values for compositional and structural descriptors, and calculated and predicted ME for all 708 materials are listed in Supporting Information. In the figure, the crowed plots indicate areas where many materials with similar compositions and structures have been reported, and where the material search has progressed relatively. However, such studies have never been conducted in empty plot areas, which correspond to unknown materials. These areas are considered risks in the context of material exploration owing to the high possibility of materials not being synthesized. Hence, the sparse plot areas might be promising in terms of both synthesizability and discovery of unknown materials. In Fig. 4, the plotted colors shift from red to blue (corresponding to a decrease in ME) as the value of structural coding increases, confirming that ME is primarily linked to structural information. Appropriate control of the crystal structure is crucial in obtaining highly conductive lithium-ion materials. As the compositional coding increased, the plot distribution became narrower, concentrating in a range from − 2 to 2 on the structural axis. Two possible reasons are suggested: (1) the range of possible crystal structures is limited with respect to chemical composition, and such a combination of composition and structure is not possible; or (2) vacancies in the map correspond to materials that researchers have not yet worked with. In any case, the materials map presented in Fig. 4 may be useful for summarizing reported research, visualizing unknown material areas, and providing a bird’s-eye view for materials researchers. In particular, the inclusion of an ME regression component in the autoencoder shown in Fig. 1 is largely beneficial in the search of high-ionic-conductivity materials, as the user can roughly evaluate the ME even in the non-plotted coordinates of the material map. Further details are discussed herein.

**Figure 5:**
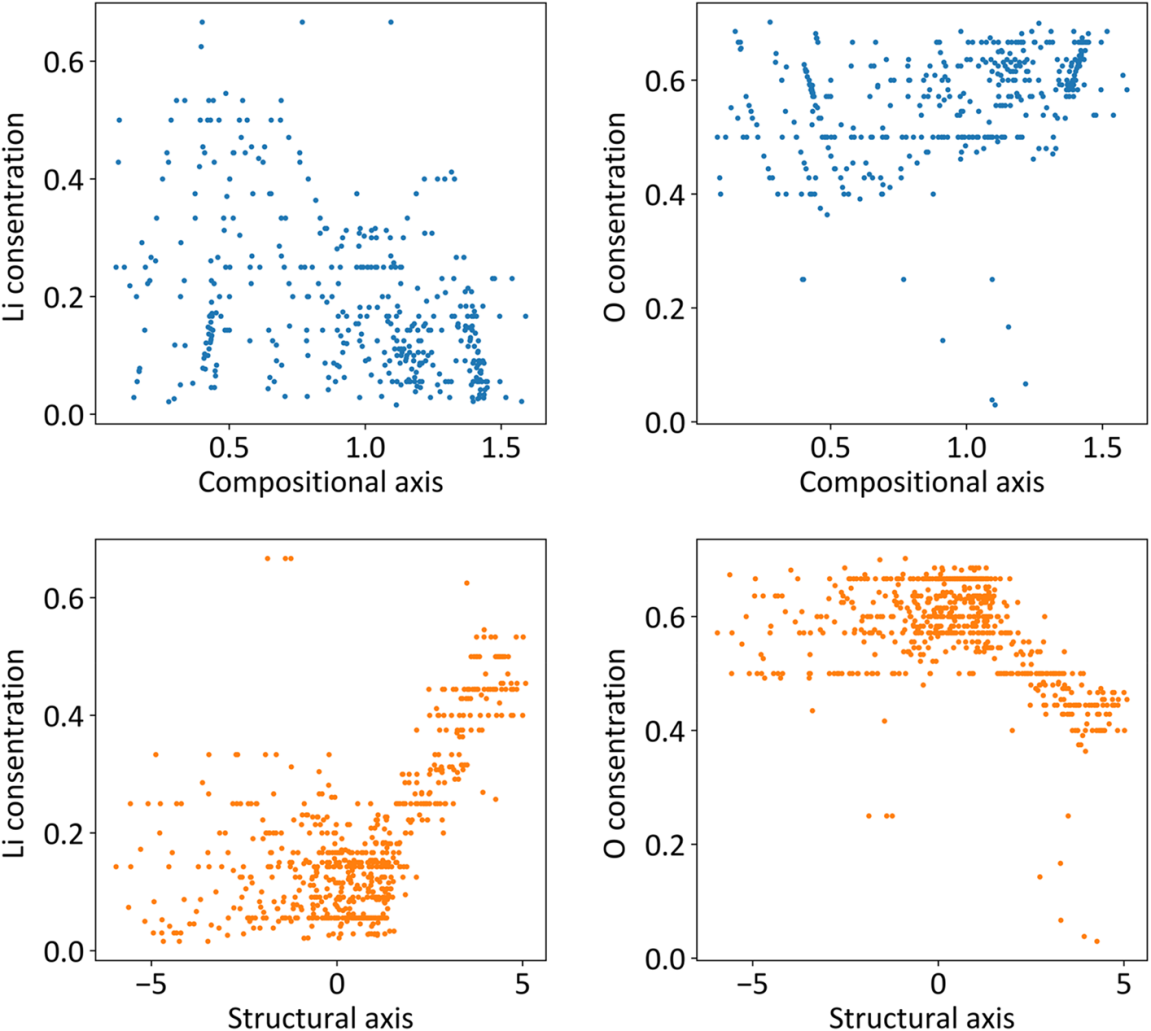
Correlation between encoded compositional variables and (**a**) Li concentration and/or (**b**) O concentration. Relationship between encoded structural encoded variables and (**c**) Li concentration and/or (**d**) O concentration.

Although the significance analysis of variables encoded by an autoencoder is generally a challenging task, we investigated factors affecting the composition and structure axes using a trial-and-error approach. The relationship between composition, structure, and high Li-ion conductivity was also considered. As a result, we infer that the lithium-to-oxygen ratio is thought to be one of the factors affecting the compositional axis. In Fig. 5, the molar ratio of (a) Li to (b) O in the composition is plotted as a function of the encoded value of the compositional descriptor *x*_comp_. At *x*_comp_ >  ~ 1.0, the molar ratio of Li tends to be <  ~ 0.3, whereas at *x*_comp_ <  ~ 1.0, it ranges from 0 to 0.6 (Fig. 5a). Similarly, the molar ratio of oxygen tends to be > 0.55 at *x*_comp_ <  ~ 1.0 (Fig. 5b), except for compounds whose molar ratio of oxygen was < 0.3. Thus, the compositional feature at *x*_comp_ >  ~ 1.0 appears to reflect relatively low Li and high O concentrations, which indicates that the proportions of lithium and oxygen are factors associated with ME. This conclusion is supported by the connection between *x*_comp_ and ME in the neural network model illustrated in Fig. 1. However, the correlation between *x*_comp_ and the ratio of Li and/or O is rather poor even at *x*_comp_ > 1; therefore, numerous other composition-related parameters must be intricately involved in the encoded value of *x*_comp_.

The relationships between the molar ratio of lithium to oxygen in the composition and the structural axes is displayed in Fig. 5c,d, respectively. A more distinct dependence on the molar ratios of Li and O is evident on *x*_struct_ as compared to *x*_comp_. Specifically, the molar ratios of Li and O are scattered at less than 0.3 and greater than 0.5, respectively, at − 5 < *x*_struct_ < 0. However, an increase in the lithium proportion and decrease in the oxygen proportion are clearly visible at *x*_struct_ > 0. This suggests that the structural axis correlates with the lithium and oxygen concentrations in the lattice at *x*_struct_ > 0. We inferred that the compositional information of the molar ratios of Li or O was indirectly extracted from the radius distribution function (RDF) of Li–O, Li–Li, etc., although these descriptors were classified as structural when *x*_struct_ > 0. In addition, regions exceeding zero on the structural axis represent clusters of materials with relatively small ME (blue distribution). Therefore, we suggest that the dense distribution of lithium and low oxygen concentration in the lattice are advantageous for achieving high ionic conduction.

**Figure 6:**
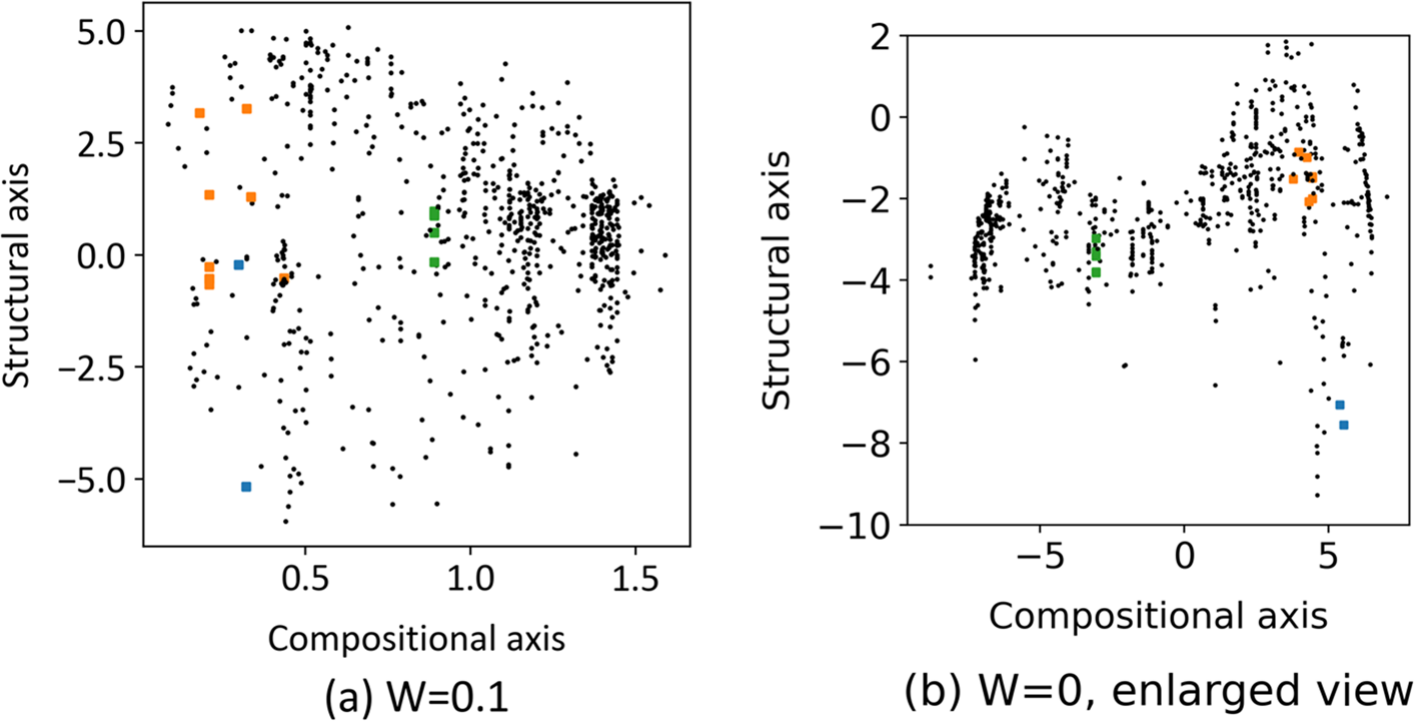
Visualization of typical Li-ion conductive materials (Perovskite, Garnet, NASICON-type materials) visualized on a materials map. Distribution of representative Li-ion conductive materials in the map at (**a**) *W* = 0.1 and (**b**) *W* = 0. Note that there are no discernible relationships between compositional/structural descriptors and ME values when W = 0.

The Li–O materials used in this study include representative host structures for fast Li-ion conductors, such as the Perovskite-type^24–26^, Garnet-type^27–31^, and NASICON-type structures^32–38^. Specific ID numbers from the Materials Project database (mp-id), chemical compositions, ME values, and encoded values for composition and structure descriptors are listed in Table 1. These data points are plotted on the material map in Fig. 6a, where each structure is relatively clustered. However, the garnet- and NASICON-type structures are located far from each other. Although differences in the materials map with respect to crystal structure (type) depend on the structural axis (vertical axis), the same structure exhibits a larger variation on the structural axis than on the compositional axis. The distributions of the Garnet and NASICON structures exhibit similar values on the structural axis, which may be simply interpreted as them being distinguished solely by composition rather than structure. One possible reason for such a counterintuitive conclusion may be the strong dependence of the structural axis on the local structure of Li and O ions, as suggested previously. In other words, the structural axis is expected to be strongly influenced by the Li coordination state and arrangement information, rather than the similarity of the host structure. For example, a significant difference in structural values was observed between Li_7_La_3_Zr_2_O_12_ and Li_7_La_3_Sn_2_O_12_, which belong to the same garnet-type material. Specifically, the lithium ions in Li_7_La_3_Zr_2_O_12_ are preferentially distributed in octahedral sites^39^, whereas those in Li_7_La_3_Sn_2_O_12_ as well as other compositions are distributed in tetrahedral sites^31^. This suggests that the structural axis is not dependent on the host structure, as classified by the Perovskite, Garnet, and NASICON types, but on the local structure around Li. This is likely because both the structural and compositional axes are associated with the target-variable ME in the neural network model, as shown in Fig. 1.

**Table 1:** Details of Perovskite, Garget, and NASICON materials in this dataset.

mp-id	Chemical formula	Structure type	ME/eV	Encoded values	
				Compositional descriptor (*x*_comp_)	Structural descriptor (*x*_*struct*_)
mp-761986	Li_5_La_3_Nb_2_O_12_	Garnet	0.983	0.207	− 0.536
mp-775628	Li_5_La_3_(SbO_6_)_2_	Garnet	0.827	0.434	− 0.517
mp-1200057	Li_7_La_3_(SnO_6_)_2_	Garnet	0.923	0.322	3.272
mp-554747	Li_5_La_3_Nb_2_O_12_	Garnet	0.543	0.207	1.342
mp-774721	Li_5_La_3_Nb_2_O_12_	Garnet	1.018	0.207	− 0.269
mp-559776	Li_5_La_3_Ta_2_O_12_	Garnet	0.529	0.334	1.300
mp-774437	Li_5_La_3_Nb_2_O_12_	Garnet	0.842	0.207	− 0.657
mp-942733	Li_7_a_3_Zr_2_O_12_	Garnet	0.861	0.178	3.172
mp-779434	Li_5_La_3_Nb_2_O_12_	Garnet	0.837	0.207	− 0.530
mp-10499	LiZr_2_(PO_4_)_3_	NASICON	0.850	0.890	0.501
mp-759280	LiZr_2_(PO_4_)_3_	NASICON	1.016	0.890	0.982
mp-773074	LiZr_2_(PO_4_)_3_	NASICON	1.261	0.890	− 0.160
mp-681439	LiZr_2_(PO_4_)_3_	NASICON	0.385	0.890	0.876
mp-541661	LiZr_2_(PO_4_)_3_	NASICON	0.865	0.890	0.918
mp-1222522	LiLa_3_Ti_4_O_12_	Perovskite	2.964	0.320	− 5.177
mp-768320	LiLa_5_Ti_8_O_24_	Perovskite	0.760	0.297	− 0.221

Materials Project ID numbers (mp-id), chemical formulae, calculated migration energies (ME), and encoded values for compositional and structural descriptors, *x*_comp_ and *x*_struct_, are listed. Note that both 1782 compositional and 858 structural descriptors are transformed into one-dimensional encoded values, referred to as x_comp_ and x_struct_, as illustrated in Fig. 1.

To confirm this, we reconstructed the material map by setting the weight of ME loss (*Loss*_*ME*_) to zero; i.e., *W* = 0. Consequently, the encoded variables became independent of ME. Figure 6b presents the resulting material map. Apparently, materials with Garnet, NASICON, and Perovskite structures are plotted as clusters and exhibit different structural encoded values in descending order of the structure axis (Fig. 6b). This suggests that structural descriptors (RDF, ADF, and Voronoi polyhedron) can capture the grouping of crystal host structures, even without considering Li-ion conductivity.

**Figure 7:**
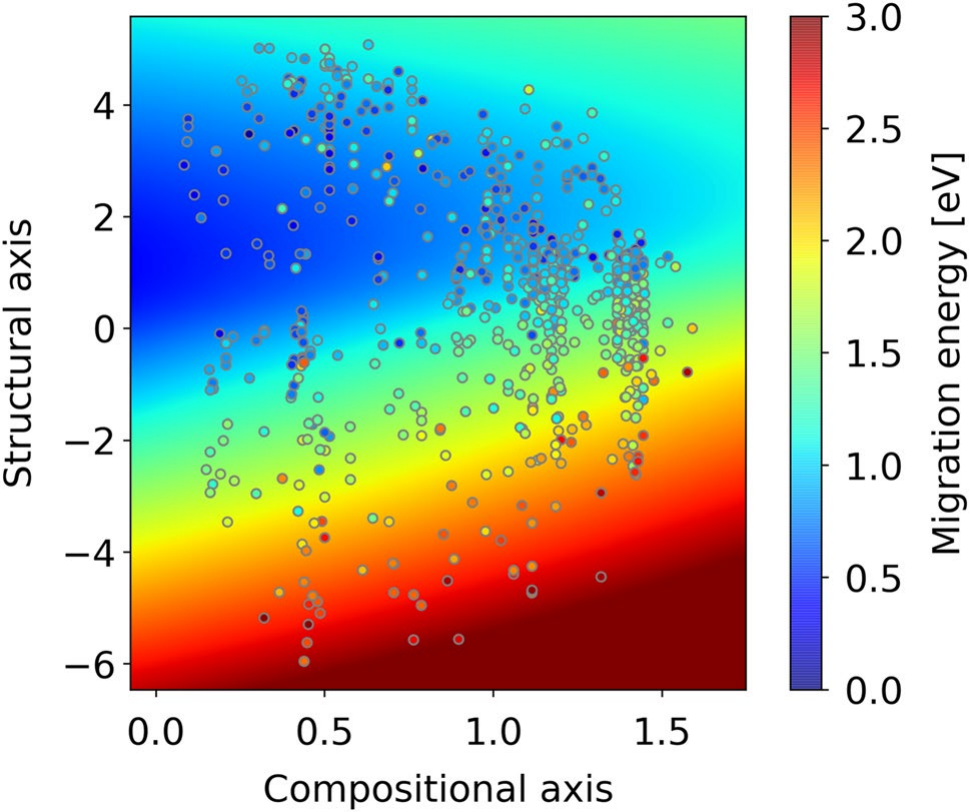
Materials map with predicted ME distribution (Background color represents predicted ME values; data points are obtained from materials simulations).

**Figure 8:**
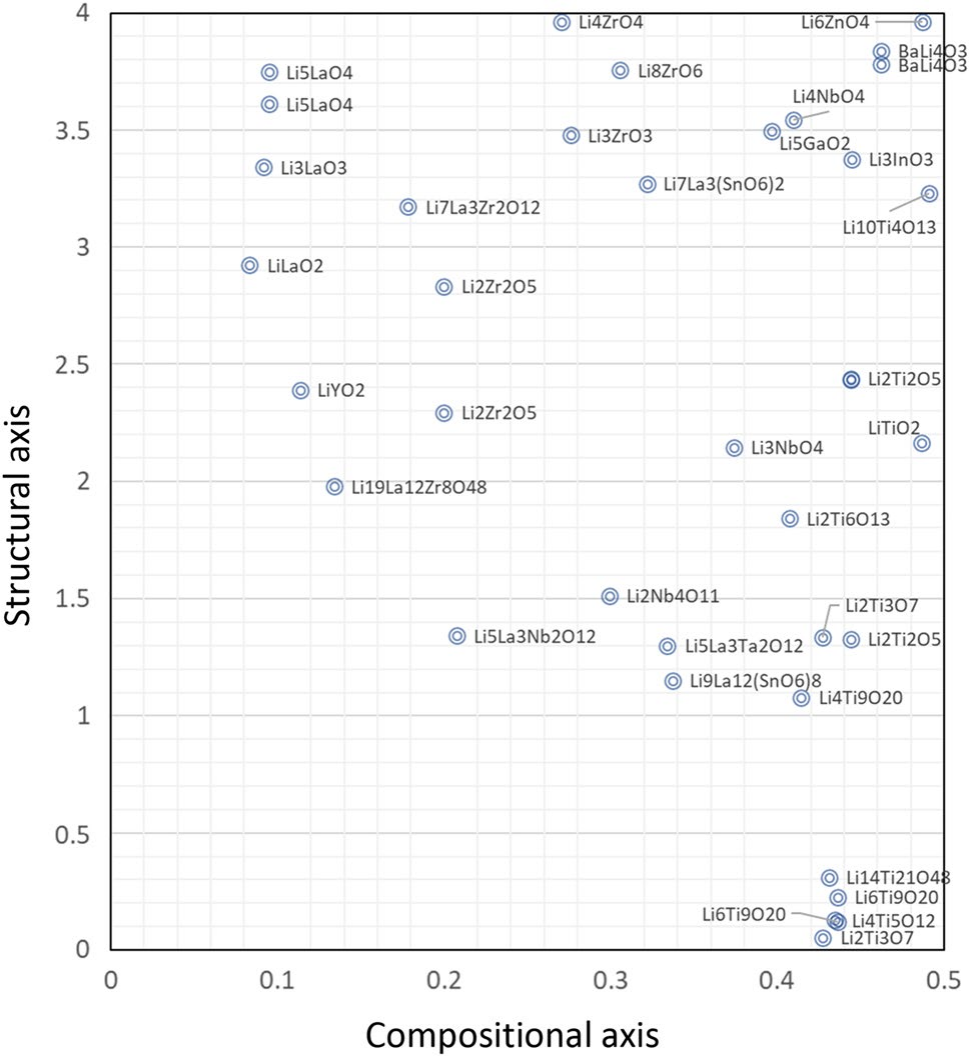
Enlarged materials map highlighting areas with anticipated high lithium conductivity and limited research. Compositions are labeled in the vicinity of their respective data points.

Figure 7 presents a heat map of the ME predicted for arbitrary coordinates on the material map using a neural network from two encoded variables to the ME (see Fig. 1). Although predictive performance for ME is low (see Fig. 3c), and, in some areas, the predicted ME values do not match the measured values shown in the plots, the map generally matches the distribution trend of the measured values. The region around *x*_comp_ ~ 0.0 and 0 < *x*_struct_ < 4 on the materials map corresponds to a lower predicted ME values, indicating higher Li-ion conductivity. Furthermore, in this region, materials registered in the database are sparsely distributed, and the possibility of synthesizing real materials is high. As shown in Fig. 5, the area is characterized by high proportions of lithium in the compounds. For example, this area prominently features garnet-type solid electrolytes, which have a higher molar ratio of Li ions than NASICON- or perovskite-type solid electrolytes. Therefore, the concentration of Li ions may be an efficient guideline for the optimization of garnet compounds by metal substitution^40^. Furthermore, several undiscovered compounds may be present in this area. Unfortunately, in principle, specifying the composition and structure for given coordinates (*x*_comp_, *x*_struct_) on the material map is difficult, as an accurate conversion from descriptors to crystal structure remains infeasible. However, a material map may inspire experienced researchers to examine the distribution of materials around the focused coordinates. For example, Fig. 8 shows the compositional distribution of materials in proximity of a relevant area, which may support the researchers’ intuitions. In addition, descriptors can be restored by decoding the input coordinates on the materials map. Although the crystal structure cannot be directly restored from descriptor information, obtaining information that can be used as material design guidelines is possible. In summary, these findings provide valuable insights for material researchers, enabling them to explore the distribution of materials, predict properties, and recover descriptors using the material map.

## Conclusion

In the field of materials informatics for inorganic materials, there are existing databases containing the data of hundreds of thousands of crystal structures. Vigorous research is underway to predict material properties using machine learning, as well as develop fast and efficient methods for optimizing functional materials. However, the vast space of registered materials in these databases suggests the presence of numerous undiscovered materials with desirable properties. Furthermore, it is difficult to systematically search a database using conventional MI methods.

In this study, we designed a materials map to visualize the distribution of known materials by using compositional and structural axes as the basis for organizing the materials. The materials map offers a quick and comprehensive overview of the current state of material exploration. For instance, areas densely populated on the materials map indicate extensive research efforts, reducing the likelihood of discovering new materials. Conversely, sparsely populated areas suggest uncertainty in material synthesis. Additionally, we successfully linked structurally and compositionally encoded values to ME values, allowing us to visualize regions on the materials map where high ionic conductivity is expected. By analyzing both the density of data points and predicted ionic conductivity values on the materials map, material researchers can make informed decisions about their next exploration area, considering results and associated risks. This approach has the potential to facilitate the discovery of materials with exceptional ionic conductivity. While other machine learning techniques such as t-Distributed Stochastic Neighbor Embedding (t-SNE)^41^ and Principal Component Analysis (PCA)^42^, can reduce descriptor dimensionality, they face challenges in decoding descriptors from arbitrarily chosen encoded values and establishing connections between encoded values and objective variables, such as migration energy in this context. In this regard, the autoencoder-based materials map we present here offers a distinct advantage. Unfortunately, although the specification of encoded variables for optimized ionic conductivity by the autoencoder-derived materials map is feasible, the reconstruction of crystal structures is technically difficult owing to the irreversibility between the structural data and their descriptors. We, therefore, believe that integrating experience and knowledge of materials researchers may be necessary for overcoming this difficulty, and we anticipate that the 2D visualization of materials information will support researchers’ in intuitively understanding material distributions.

### Supplementary Information

Supplementary Information 1.

Supplementary Information 2.

## Data Availability

All the input and output data are available in the Supporting Information.
