# Supplementary material for: Drawing a materials map with an autoencoder for lithium ionic conductors
Source: Sci Rep. 2023 Oct 5;13:16799. doi: 10.1038/s41598-023-43921-1 (PMC10556005; doi:10.1038/s41598-023-43921-1)
Supplement: Supplementary file 2 — Supplementary Information 2. [file 41598_2023_43921_MOESM2_ESM.docx]

**Supporting Information for**

**Drawing a materials map with an autoencoder for lithium ionic conductors**

Yudai Yamaguchi^1)^, Taruto Atsumi^1)^, Kenta Kanamori^2)^, Naoto Tanibata^1)^, Hayami Takeda^1)^, Masanobu Nakayama^1)*^, Masayuki Karasuyama^2)^, Ichiro Takeuchi^2-4)^

1. Department of Advanced Ceramics, Nagoya Institute of Technology, Gokiso, Showa, Nagoya, Aichi 466-8555, Japan
2. Department of Computer Science, Nagoya Institute of Technology, Gokiso-cho, Showa-ku, Nagoya, Aichi 466-8555, Japan
3. RIKEN Center for Advanced Intelligence Project, 1-4-1 Nihonbashi, Chuo-ku, Tokyo, 103-0027, Japan
4. Faculty of Engineering, Nagoya University, Furo-cho, Chikusa-ku, Nagoya, Aichi, 464-8601, Japan

*Corresponding author: [masanobu@nitech.ac.jp](mailto:masanobu@nitech.ac.jp)

The Supporting Information file attached as Comma separated values (CSV) format contains composition and structure descriptors in the form of a histogram for crystal structures extracted from the Materials Project^1,2^. Figure S1 shows the details on how to construct descriptors. The lithium ion migration energies deduced by the BVFF method^3–5^, as well as the compressed values of the compositional descriptors (encoded_composition) and structural descriptors (encoded_structure) encoded by the autoencoder, and the migration energy (predicted_ME) are also listed. The first line in the file corresponds to the descriptor labels. The following sections provide notationa and description on the consisting compositional and structural descriptors.

Table S1 lists the various properties derived from the compositions used in this study and their abbreviations. The conditions for histogramming these descriptors (minimum, maximum, number of separations and standard deviation of broadening parameters) are also listed in Table S2. Furthermore, the histogrammed differences and products of the various property values between the different constituent elements are also added as descriptors. The maximum and minimum values of the histograms of the differences and products are the maximum and minimum values listed in Table S2 multiplied by two or the product, respectively. The labels of the descriptors corresponding to the differences and products were denoted as EN-EN or ENEN for electronegativity (EN). The number attached to the label indicates the index number of the bin. The composition descriptors described above were generated by in-house software.^6,7^

Table S3 shows the structural descriptors for the geometric arrangement of the ions. In this study, the Radial distribution function (RDF), which histograms the bond lengths and bond angles, and the Angular distribution function (ADF), which histograms the features of the figures of the voronoi partitions of the ions constituting the crystal, and the The Voronoi splitting was done using the voro++ software^8–10^.Table S4 shows the minimum, maximum, number of separations and standard deviation parameters of the Gaussian broadening of the histograms.


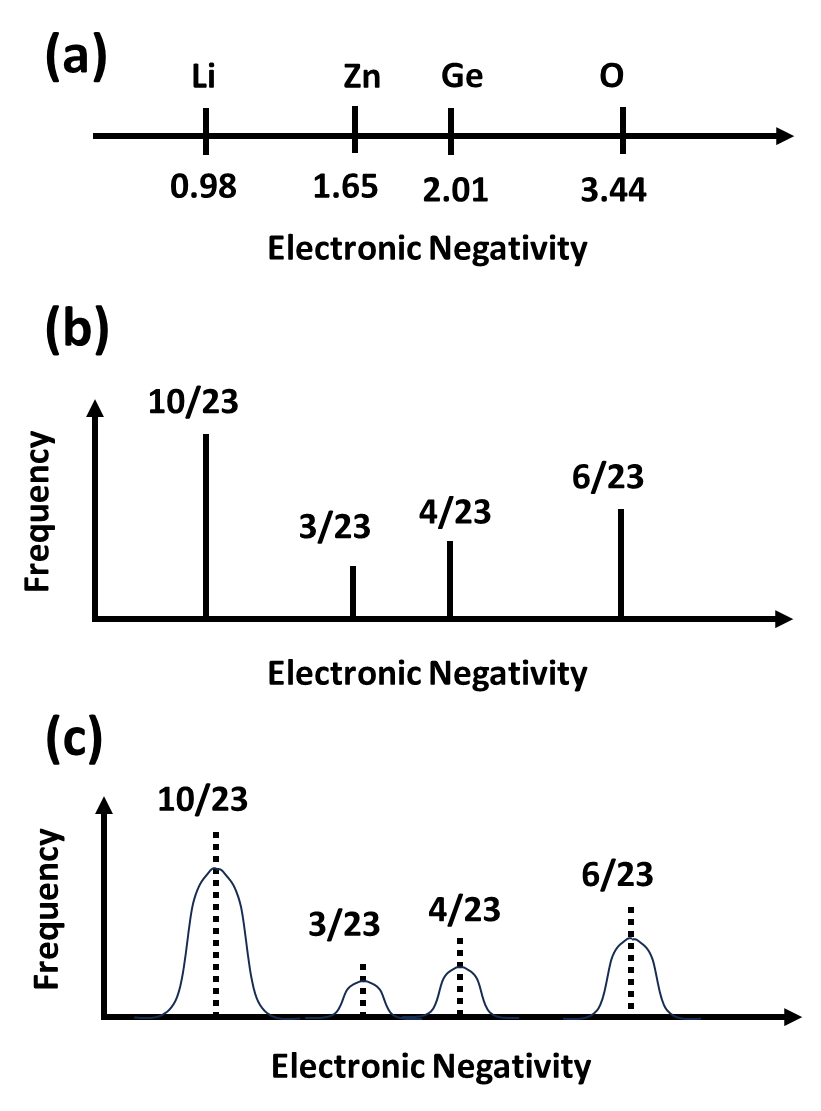


**Figure S1** Schematic diagram of making descriptosr in the form of a histogram from compositional and structural information^6^. Descriptors from the electronegativity information of the constituent elements in Li_10_Zn_3_Ge_4_O_6_ is shown as an example. (a) The horizontal axis is the electronegativity of the constituent elements (Li, Zn, Ge and O) and (b) the vertical axis is the composition ratio of each element in the histogram. The composition ratios are normalized to be 1 as a summation of all the consisting atoms in the given composition. (c) The histograms are processed by Gaussian broadening.

**Table S1**  Extracted element derived properties or parameters for compositional descriptors used in this study.

| Label | Description |
| --- | --- |
| AN | Atomic number |
| EN | Electronic negativity by Allred and Rochow^11^ |
| MP | Melting point of the simple substance^12^ |
| PN | Periodic number |
| PG | Periodic group |
| MN | Mendeleyev number |
| AW | Atomic weight |
| AR | Atomic radius^13^ |
| IR | Ionic radius^14^ |
| CoR | Covalent radius^15^ |
| CrR | Crystal radius^16^ |
| spdf | Label whether the element belongs to s, p, d, f-blocks in the periodic table (s,p,d,f)=(1,2,3,4) |

**Table S2**  Parameters for construction nof histogram for compositional descriptors. The colomun “Label” corresponds to Table S1.

| Label | # of bins | Max | Min | Broadening factor |
| --- | --- | --- | --- | --- |
| AN | 102 | 120 | -24 | 4.8 |
| EN | 50 | 5 | -10 | 0.2 |
| MP | 50 | 5000 | -1000 | 200 |
| PN | 20 | 10 | -2 | 0.4 |
| PG | 20 | 20 | -4 | 0.8 |
| MN | 102 | 120 | -24 | 4.8 |
| AW | 50 | 400 | -80 | 16 |
| AR | 50 | 3.2 | -0.64 | 0.128 |
| IR | 50 | 3.2 | -0.64 | 0.128 |
| CoR | 50 | 3.2 | -0.64 | 0.128 |
| CrR | 50 | 3.2 | -0.64 | 0.128 |
| spdf | 4 | 4 | 1 | 0 |

**Table S3** Description of structure descriptors used in this study.

| Label | Description |
| --- | --- |
| RDF_X1 | Radial distribution function, X1 is the bonding pair and Li-O, Li-Li and O-O, which are common to all samples, are selected. all extracts the radial distribution function for all bonding pairs, regardless of element. (unit Å) (unit Å) |
| ADF_X2 | Angular distribution function, X2 indicates the combination of bond angles. For example, Li-O-Li corresponds to the bond angle formed by two Li ions bonding to oxygen. The elements of interest were Li and O, which are common to all samples. The bonds were extracted with cut-off length = 2.8 Å. (unit deg.). |
| g.voronoi_X3 | Histogram of the number of edges of the voronoi polyhedron generated by the voronoi division of space in the lattice for ion X3, where X3 is Li and O and cation/anion were chosen. |
| s.voronoi_X3 | Histogram of the number of faces of the Voronoi polyhedron. Details are the same as in g.voronoi_X3. |
| w.voronoi_X3 | Histogram of the number of vertices of the Voronoi polyhedron. Details are the same as in g.voronoi_X3. |

**Table S4** Parameters for construction of histogram for structure descriptors. The colomun “Label” corresponds to Table S3.

| Label | # of bins | Max | Min | Broadening factor |
| --- | --- | --- | --- | --- |
| RDF_X1 | 20 | 6 | 0 | 4.8 |
| ADF_X2 | 60 | 180 | 0 | 5 |
| g.voronoi_X3 | 50 | 90 | -10 | 50 |
| s.voronoi_X3 | 30 | 50 | -10 | 30 |
| w.voronoi_X3 | 40 | 70 | -10 | 40 |

**References**

1. Ong, S. P. *et al.* The materials project. *Mater. Proj.* (2013).

2. Jain, A. *et al.* Commentary: The Materials Project: A materials genome approach to accelerating materials innovation. *APL Mater.* **1**, 11002 (2013).

3. Adams, S. & Rao, R. P. High power lithium ion battery materials by computational design. *Phys. status solidi* **208**, 1746–1753 (2011).

4. Chen, H. & Adams, S. Bond softness sensitive bond-valence parameters for crystal structure plausibility tests. *IUCrJ* **4**, 614–625 (2017).

5. Nakayama, M., Kimura, M., Jalem, R. & Kasuga, T. Efficient automatic screening for Li ion conductive inorganic oxides with bond valence pathway models and percolation algorithm. *Jpn. J. Appl. Phys.* **55**, 5–9 (2016).

6. Jalem, R. *et al.* A general representation scheme for crystalline solids based on Voronoi-tessellation real feature values and atomic property data. *Sci. Technol. Adv. Mater.* **19**, 231–242 (2018).

7. compositional-histogram-descriptor. https://github.com/NakayamaLab-NITech/composional-histogram-descriptor

8. Rycroft, C. H. VORO++: A three-dimensional Voronoi cell library in C++. *Chaos An Interdiscip. J. Nonlinear Sci.* **19**, 41111 (2009).

9. Rycroft, C. H., Grest, G. S., Landry, J. W. & Bazant, M. Z. Analysis of granular flow in a pebble-bed nuclear reactor. *Phys. Rev. E* **74**, 21306 (2006).

10. voro++. https://github.com/chr1shr/voro.

11. Allred, A. L. & Rochow, E. G. A scale of electronegativity based on electrostatic force. *J. Inorg. Nucl. Chem.* **5**, 264–268 (1958).

12. Kaye, G. W. C. & Lavy, T. H. *Macmillan’s Chemical and Physical Data*. (Longman, 1993).

13. Clementi, E. & Raimondi, D. L. Atomic Screening Constants from SCF Functions. *J. Chem. Phys.* **38**, 2686–2689 (2004).

14. Slater, J. C. Atomic Radii in Crystals. *J. Chem. Phys.* **41**, 3199–3204 (2004).

15. Cordero, B. *et al.* Covalent radii revisited. *Dalt. Trans.* 2832–2838 (2008) doi:10.1039/B801115J.

16. Shannon, R. D. Revised effective ionic radii and systematic studies of interatomic distances in halides and chalcogenides. *Acta Crystallogr. Sect. A* **32**, 751–767 (1976).
